# Comprehensively analysis of splicing factors to construct prognosis prediction classifier in prostate cancer

**DOI:** 10.1111/jcmm.17849

**Published:** 2023-08-09

**Authors:** He Zhang, Jianfei Tian, Sixin Ren, Baoai Han, Ruinan Tian, Xiaoyan Zuo, Hui Liu, Zhiyong Wang, Yanfen Cui, Liming Liu, Hui Guo, Fei Zhang, Ruifang Niu

**Affiliations:** ^1^ Public Laboratory Tianjin Medical University Cancer Institute and Hospital, National Clinical Research Center for Cancer Tianjin China; ^2^ Key Laboratory of Cancer Prevention and Therapy Tianjin China; ^3^ Tianjin's Clinical Research Center for Cancer Tianjin China

**Keywords:** alternative splicing, cell cycle, prognosis, prostate adenocarcinoma, splicing factor

## Abstract

Splicing factors (SFs) are proteins that control the alternative splicing (AS) of RNAs, which have been recognized as new cancer hallmarks. Their dysregulation has been found to be involved in many biological processes of cancer, such as carcinogenesis, proliferation, metastasis and senescence. Dysregulation of SFs has been demonstrated to contribute to the progression of prostate cancer (PCa). However, a comprehensive analysis of the prognosis value of SFs in PCa is limited. In this work, we systematically analysed 393 SFs to deeply characterize the expression patterns, clinical relevance and biological functions of SFs in PCa. We identified 53 survival‐related SFs that can stratify PCa into two de nove molecular subtypes with distinct mRNA expression and AS‐event expression patterns and displayed significant differences in pathway activity and clinical outcomes. An SF‐based classifier was established using LASSO‐COX regression with six key SFs (BCAS1, LSM3, DHX16, NOVA2, RBM47 and SNRPN), which showed promising prognosis‐prediction performance with a receiver operating characteristic (ROC) >0.700 in both the training and testing datasets, as well as in three external PCa cohorts (DKFZ, GSE70769 and GSE21035). CRISPR/CAS9 screening data and cell‐level functional analysis suggested that LSM3 and DHX16 are essential factors for the proliferation and cell cycle progression in PCa cells. This study proposes that SFs and AS events are potential multidimensional biomarkers for the diagnosis, prognosis and treatment of PCa.

## 1 INTRODUCTION

Splicing factors (SFs) are mainly RNA‐binding proteins that control the alternative splicing (AS) of RNAs.^1^ AS is a post‐transcription process in which the precursor RNAs are processed to become their mature forms.^2^ In this process, one or more isoforms can be produced from the same genes by combining different exons, which greatly increases the diversity of the gene products.^3^ It is believed that SF‐controlled AS events modify approximately 90% of human genes and constitute one of the essential mechanisms regulating various biological processes such as cell proliferation, embryonic morphogenesis, immune response and cancer progression.^1^, ^4^ AS includes seven types of alternative splicing: alternate acceptor site (AA), alternate donor site (AD), alternate promoter (AP), alternate terminator (AT), exon jump (ES), exon mutual exclusion (ME) and intron retention (RI).^5^ It has been proposed that dysregulation or mutation of SFs can promote the occurrence of cancers and confer advantages for the tumour cells, including uncontrolled proliferation, stemness, anti‐apoptosis and evasion of immune surveillance.^2^, ^6^ Researchers have found that some genes can switch between ‘oncogenic’ and ‘tumour‐suppressive’ states via AS.^7^ These findings suggest that SFs could serve as biomarkers or therapeutic targets for cancer management.

Prostate cancer (PCa) is one of the major life‐threatening diseases worldwide.^8^, ^9^ Complex coordinated molecular and cellular events are known to contribute to the carcinogenesis, proliferation and metastasis of PCa.^10^ Recently, accumulated studies suggest that SFs are involved in many aspects of PCa. The dysregulation of several key SFs, such as HNRNPL (Heterogeneous Nuclear Ribonucleoprotein L), MALAT1 (Metastasis Associated Lung Adenocarcinoma Transcript 1), SRRM3 (Serine/Arginine Repetitive Matrix 3) and SRRM4 (Serine/Arginine Repetitive Matrix 4), has been shown to contribute to the progression of PCa.^11^, ^12^, ^13^ Isoform switching of the androgen receptor (AR) has also been found deeply involved in the progression of PCa.^14^ Alternative splicing of NF‐YA (Nuclear Transcription Factor Y Subunit Alpha) has been demonstrated to promote PCa aggressiveness and represents a new molecular marker for the clinical stratification of patients.^15^ However, a comprehensive analysis of the prognostic value of SFs in PCa is limited.

In this study, we systematically analysed 393 SFs to deeply characterize their expression patterns, prognostic value and biological functions in PCa. We identified two de novo molecular subtypes with distinct mRNA expression patterns, signalling pathway activity, AS patterns and prognoses. Furthermore, we constructed an SF‐classifier based on six key splicing factors can accurately predict the clinical outcomes of PCa patients, including BCAS1 (Brain Enriched Myelin Associated Protein 1), LSM3 (LSM3 Homologue, U6 Small Nuclear RNA And MRNA Degradation Associated), DHX16 (DEAH‐Box Helicase 16), NOVA2 (NOVA Alternative Splicing Regulator 2), RBM47 (RNA Binding Motif Protein 47) and SNRPN (Small Nuclear Ribonucleoprotein Polypeptide N). Finally, our cell‐level experiments demonstrated that DHX16 and LSM3 play essential roles. This work provides valuable information for the diagnosis and prognosis of PCa. The newly identified SF subtypes and SF‐classifier illustrate the important roles of SFs in cancer progression.

## 2 MATERIALS AND METHODS

### 2.1 Data collection and preprocessing

The Cancer Genome Atlas (TCGA) RNAseq data, in both Fragments Per Kilobase of exon model per Million mapped fragments (FPKM) and RAW count formats, were obtained from the TCGA data portal. The FPKM data were transformed into transcripts per million (TPM) for downstream analysis. The batch effect of the TCGA RNAseq data was examined using the TCGA Batch Effects Viewer (https://bioinformatics.mdanderson.org/public‐software/), and no significant batch effect was identified. The DKFZ (Deutsches Krebsforschungszentrum) dataset (Whole genome and transcriptome sequencing of tumour/normal pairs from 292 prostate cancer patients) was downloaded from cBioPortal (https://www.cbioportal.org/study/summary?id=prostate_dkfz_2018).^16^ GSE70769 (Prostate cancer stratification using molecular profiles, Cancer Research UK Cambridge Institute) and GSE21035 (Agilent 244K aCGH data for human primary and metastatic prostate cancer samples, Memorial Sloan‐Kettering Cancer Center, SMKCC) were downloaded from the GEO database (https://www.ncbi.nlm.nih.gov/geo).

The TCGA‐SplicingSeq data were downloaded from the TCGA SplicingSeq database (https://bioinformatics.mdanderson.org/TCGASpliceSeq/index.jsp). The RAW SplicingSeq data were preprocessed to omit any alternative splicing events with an ‘NA’ value in over 30% of all cases. The Percent Spliced In (PSI) index, an intuitive ratio for quantifying splicing events ranging from 0 to 1, was calculated for seven types of AS patterns: ES, ME, RI, AP, AT, AD and AA. The data were further processed using the ‘impute’ R package to fill in missing values using the K‐nearest neighbours (KNN) method. The imputed PSI data were filtered based on the standard deviation (SD), and AS events with an SD of less than 0.15 were excluded from the study.

The somatic mutation data of PCa samples were obtained from the TCGA data portal by using GDC tools (http://portal.gdc.cancer.gov/). The ‘maftools’ R package was used to process and visualize the data.^17^

The clinicopathological information of the TCGA data was obtained from the Xnea database (https://xena.ucsc.edu/). For the TCGA‐PRAD dataset, given the consideration that overall survival time requires a longer follow‐up period and may not promptly reflect the disease progression, we used the Progression‐Free Interval (PFI) as a more accurate measurement. The PFI data were defined according to the method outlined in the article by Jianfang Liu et al.^18^ For GSE70769 and GSE21035 datasets, the clinicopathological was downloaded from GEO database. The clinical data were manually curated and cases with incomplete survival data were omitted from the downstream analysis. For the DKFZ, GSE70769 and GSE21035 datasets, we used Biochemical Relapse‐Free Survival (BCR) as a prognosis indicator. The detailed clinicopathological information is provided in Table S1.

### 2.2 Analysis of the expression and survival landscapes of SFs and AS‐events

The gene list of SFs was obtained from a previous pan‐cancer study conducted by Seiler et al.^3^ SFs from the list that did not match TCGA‐PRAD data were omitted from this study. A total of 393 SFs, shown in Table S2, were subjected to the downstream analysis. For differential expression analysis and univariable COX analysis of SFs, the TPM data were log2 transformed, the *survival* R package was used to perform the univariable COX analysis, and a strict cut‐off (*p* < 0.001) was implemented to define the survival‐significant SFs. For differential expression analysis of SFs between tumour and tumour tissues, the RAW count value of mRNA expression was used and analysed using the *DESeq2* package.^19^ For AS events, the PSI value of AS data was directly subjected to differential expression calling between subtypes. For univariable COX analysis of AS events, the PSI values were Z‐normalized before analysis. The selected key SFs and AS events were also subjected to multivariable‐COX analysis using the *survival* package.

### 2.3 ConsensusClusterPlus analysis and principal component analysis

The *ConsensusClusterPlus* package was used to explore the molecular subtypes in TCGA‐PRAD dataset using the 53 survival‐significance SFs.^20^ The parameters for the analysis were set as follows: reps = 50, clusterAlg = km, distance = euclidean. The optimal cluster number was chosen by manually checking the consensus matrix and consensus cumulative distribution function (CDF) output, with two clusters selected for downstream analysis. For principal component analysis (PCA), the mRNA expression data were utilized (log2‐transformed TPM for TCGA‐PRAD; PSI value for AS‐events) and analysed using the *pca3d* package in R.

### 2.4 Construction and validation SFs based‐PIF‐classifier

For the construction of a splicing factors‐based risk classifier for the prediction of PSI, we used the TCGA‐PRAD dataset for the LASSO‐COX model construction and then the classifier was verified by three external PCa cohorts. In brief, patients were randomly divided into training and testing groups. The 53 survival‐significant SFs were subjected to LASSO regression (using the *glmnet* R package) to eliminate the false‐positive parameters caused by overfitting. Finally, a multivariable Cox analysis was used to calculate the Hazard ratio and coefficients, thereby generating the prognosis model. The calculation formula is as follows:

$$\text{RiskScore}=\sum_{i=1,2,3,i\;}\text{coefficient}\;\left(\mathrm{SFi}\;\right)\times \text{expression}\left(\mathrm{SFi}\right),$$

In this formula, the SFi represents the identifier of the *i*th selected SF. The coefficient (SFi) is the COX‐regression coefficient, estimated by the Cox proportional risk regression analysis for a specific SFi. The RiskScore was then calculated as a measurement of the prognostic risk of each PCa patient. The predictive power of the constructed classifier was then verified using an internal testing dataset and three independent external PCa cohorts (DKFZ, GSE70769 and GSE21035) via Kaplan–Meier analysis and time‐dependent receiver operating characteristic (ROC) analysis. Models with a *p* value <0.05 and an area under the curve (AUC) >0.700 were considered to have acceptable predictive power.

### 2.5 Gene set variant analysis and immune cell infiltration analysis

The gene set variation analysis (GSVA) was performed using TPM value of TCGA‐PRAD RNAseq data using *GSVA* package. The significantly enriched signalling pathways between the two identified SF subtypes were identified using the limma package, which used the GSVA score of each TCGA‐PRAD case. The HALLMARKS50 and KEGG Pathway gene sets were used in the analyses.

The infiltration levels of 22 types of immune cells were estimated using the CIBERSORTx program (https://cibersortx.stanford.edu/), with bulk tumour RNA‐Seq data as the input.^21^ CIBERSORTx was run in relative mode, with batch effect correction applied in S‐mode, using RNA‐Seq TPM data and the built‐in LM22 signature matrix.

### 2.6 Identification of key AS‐event and construction of AS‐risk model

The key AS events were identified following a previously described method.^5^ Briefly, 57 AS events were selected based on the following criteria^1^: AS events that were dysregulated in the 2 SF subtypes with a Wilcoxon test FDR <0.01 and |log2FC| > 0.3^2^; AS events that were significantly associated with PFI with a univariable COX *p* < 0.001. These 57 AS events were then subjected to LASSO‐COX regression analysis to screen for the most prognostically significant AS events. Six key AS events were identified, leading to the construction of an AS‐risk model as follows:

$$\text{ASRiskscore}=\sum_{i=1,2,3,i\;}\text{coefficient}\;\left(\mathrm{ASi}\;\right)\times \mathrm{PSI}\left(\mathrm{ASi}\right),$$

The ASi represents the identifier of the *i*th selected AS event. The coefficient (ASi) is the Cox‐regression coefficient estimated from the Cox proportional risk regression analysis for a specific ASi. The predictive power of the AS‐risk model was then analysed using Kaplan–Meier and time‐dependent ROC analyses. Models that exhibited a log‐rank *p* value <0.05 and an AUC >0.700 were considered to have acceptable predictive power.

### 2.7 Cell culture

Human prostate cancer cell lines PC3 and DU145 were purchased from the American Type Culture Collection (ATCC, Manassas, VA, United States). The cells were maintained in a humidified incubator containing 5% CO_2_ at 37°C. PC3 and DU145 cells were cultured in RPMI‐1640 medium and supplemented with 10% foetal bovine serum (HyClone), 100 U/mL penicillin (HyClone) and streptomycin (HyClone). The cells used in this work were routinely checked by morphological observation and tested for mycoplasma contamination.

### 2.8 Small interfering RNA transfection and quantitative real‐time PCR analysis

The small interfering RNA (siRNA) against NOVA2, LSM3 and DHX16 were synthesized by GenePharma (Tianjin, China). The siRNA transfection was performed using Lipofectamine RNAiMax (Thermo Fisher Scientific) following the manufacturer's instructions. The siRNA sequences used in this study are shown as follows (5′‐3′): siNC: UUCUUCGAACGUGUCACGUTT; siDHX16: GGCAGGAGCUCAAAUAUAATT; siLSM3: AAUAUUCCAAUGCUCUUUGTT; siNOVA2: GCUUAACACGCUGGCAAGUTT.

For quantitative real‐time PCR (qRT‐PCR) analysis, total RNA was extracted using Trizol (Life Technologies, Carlsbad), and the cDNA was prepared using HiScript II Q RT SuperMix for qPCR (Vazyme, China). The qRT‐PCR reaction was performed using AceQ qPCR SYBR Green Master Mix (Vazyme, China) following the manufacturer's instructions. All qRT‐PCRs were run in triplicates and normalized with the β‐actin housekeeping gene. The primers for qRT‐PCR experiments are shown as follows (5′‐3′): NOVA2, F: CCCGCTGAGGATGTGGATAC, R: CATGGTTAGAGGAGCCGTCC; LSM3, F: AGCCCCTCTCAACCTCAGTA, R: CATGGTTAGAGGAGCCGTCC; DHX16, F: CAGCACCAATGCAAACCCTC, R: TCCCGAGAGAGGTCAAGGAG; β‐actin, F: CTGGGTGTTGAAGGTCTC, R: CAGAGCAAGAGAGGCATCC, which were designed by the Primer Blast program.

### 2.9 Cell proliferation assay colony formation assay, cell cycle analysis and cell apoptosis analysis

The cell proliferation of PCa cell lines was examined by using Cell Count Kit 8 (CCK8) assay as previously described.^22^ Briefly, the PC3 and DU145 were seeded in 96‐well plates in at least triplicated at a density of 2000 (PC3) or 1500 (DU145) cells/well. The cell proliferation was determined by adding 100 μL of CCK8 reagent (Bimake, Houston, TX, USA) into each well and incubating at 37°C for 2–3 h, and then, the absorbance was measured at 450 nm. For colony formation analysis, 500 DU145 or PC3 cells were seeded in 6‐well plates and cultured for 14 days with routine observation. Then, the cells were washed with ice‐cold PBS, fixed with methanol and stained using crystal violet staining buffer.

For the cell cycle assay, cells were fixed with 75% ethanol at 4°C overnight. The cells were then centrifuged, washed with PBS and stained with propidium iodide (50 μg/mL) in the presence of RNase A for 10 min at 25°C. For cell apoptosis assay, the PC3 and DU145 cells were transfected with siRNAs for 72 h and stained using the Annexin V–FITC/propidium iodide (PI) Apoptosis Detection Kit (Vazyme, China), following the manufacturer's instructions. The cells were analysed using a flow cytometer, and the data were processed by FlowJo V10 (for apoptosis assay) and Modfit (for cell cycle analysis) software.

### 2.10 CRISPR/CAS9 screen data analysis

The CRISPR/CAS9 screen data were analysed using the Project Score database (https://score.depmap.sanger.ac.uk). We used the fitness score, a quantitative measure of the cell viability effect elicited by CRISPR‐Cas9 mediated cell inactivation, as the metric to evaluate the significance of genes in cell survival and proliferation. This is based on Bayes Factor values computed using BAGEL on CRISPRcleanR corrected gene depletion fold changes.^23^ Values are scaled to a 5% false discovery rate threshold from classifying reference essential and non‐essential genes.

### 2.11 Western blot analysis

The western blot analysis was carried out as previously described.^24^ The antibodies used in this study were summarized as follows: Cyclin B1 (#12231s, CST, Beverly, MA, USA), Cyclin D1 (ab134175, Abcam, Cambridge, MA, USA), Cyclin E1 (sc‐247, Santa Cruz Biotechnology, Santa Cruz, CA, USA), β‐actin (A1978, Sigma‐Aldrich, MO, USA), HRP‐linked Anti‐Rabbit IgG (#1706515, BIO‐RAD) and HRP‐linked Anti‐Mouse IgG (#1706516, BIO‐RAD).

### 2.12 Statistical analysis

All statistical analyses were performed using R software (version 4.2.1) or GraphPad Prism (version 8.0). The Wilcoxon test or Kruskal–Wallis test was used to evaluate the distribution differences among variables. Kaplan–Meier survival curve analysis and the log‐rank test were used to analyse OS. *p* < 0.05 was considered statistically significant. The R packages *ggplot2*, *ggpubr* and *survivalROC* were used to generate the time‐dependent ROC curves. The AUC value greater than or equal to 0.70 was regarded as having significant predictive value, and an AUC value greater than or equal to 0.65 was regarded as having an acceptable predictive value.

## 3 RESULTS

### 3.1 Flowchart of this Study

**FIGURE 1:**
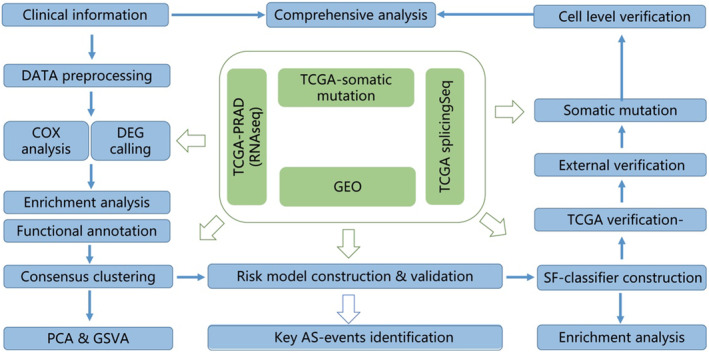
Flowchart of this study. The workflow of this study. The clinicopathological information, mRNA expression, splicing and somatic mutation data were subjected to the following analyses as the figure mentioned.

The design, data processing and analysis in this study are depicted in Figure 1. First, we acquired TCGA‐RNA‐Seq, TCGA‐Somatic mutation, TCGA‐SplicingSeq and GEO‐mRNA expression data for PCa. These datasets underwent preprocessing and quality control measures and were analysed according to the various modules shown in Figure 1. Moreover, we downloaded and manually reviewed clinicopathological data associated with each dataset to exclude cases with incomplete follow‐up information or missing essential clinical details for downstream analyses.

We initially performed a COX analysis combined with differential expression analysis to identify the most clinically significant splicing factors (SFs). We then subjected these genes to functional annotation and enrichment analyses. Following this, we conducted consensus clustering analyses to identify consensus molecular subtypes (defined as SF subtypes) based on the selected splicing factor genes. We further delineated the differences in molecular characteristics of these SF subtypes through PCA and GSVA analyses. Key alternative splicing events were then identified based on the differentially expressed AS events between the two SF subtypes.

The SF‐classifier (risk model) was built using survival‐significant SFs in the TCGA internal training and testing dataset and further validated using three independent PCa cohorts. Somatic mutation analysis was carried out to identify key mutation events in the classifier‐predicted high‐ and low‐risk groups based on TCGA data. Finally, cell‐level experiments, including proliferation assay, cell cycle assay, cell apoptosis assay and western blotting, were conducted to confirm the biological functions of the identified SFs (the SFs used to construct the classifier).

### 3.2 Survival‐related SFs predict two molecular subtypes with different prognoses

**FIGURE 2:**
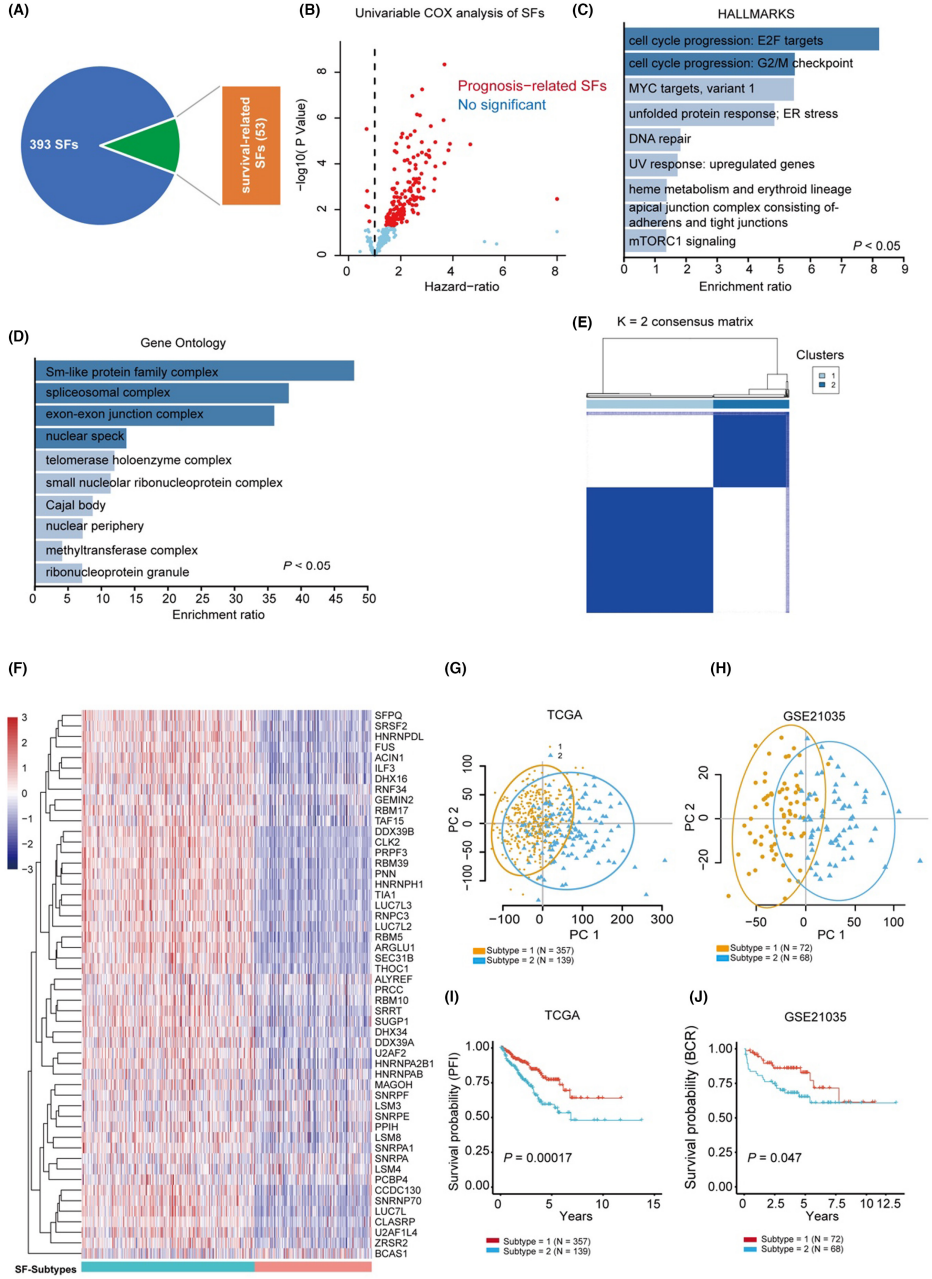
Survival‐related SFs predict two molecular subtypes with different prognosis. (A) The pie plot showed the univariable‐cox analysis result of 393 SFs, 53 survival‐significant SFs were identified. (B) The volcano plot showed the distribution of Hazard ratio and *p* values of all analysed SFs in TCGA‐PRAD dataset; the red dots indicated the survival‐significance SFs. (C, D) Overrepresentation analysis of the survival‐significant SFs using HALLMARKS and Gene Ontology Cellular Compartment genesets, respectively. (E) The consensus map showed two subtypes in PCa using the survival‐significant SFs. (F) Heatmap showed the mRNA expression pattern of 53 survival‐significant SFs in TCGA‐PRAD dataset. (G, H) Principal component analysis (PCA) of tumour samples in TCGA‐PRAD (G) and GSE21035 (H), the SF subtybes identified by ConsensusClusterPlus were marked as blue triangles and yellow circles. (I, J) Kaplan–Meier analysis of biochemical‐free survival (BCR) of two clusters of PCa patients in TCGA‐PRAD (I) and GSE21035 (J) datasets (log‐rank test).

To screen out the survival‐related SFs, we first calculated the COX hazard ratio of all involved SFs using the TCGA‐PRAD dataset. Interestingly, we found most of the survival‐significance SFs have a >1 hazard ratio, which suggests that high levels of specific SFs may promote the progression of PCa (Figure 2A,B). A total of 53 survival‐related SFs were selected based on the results of the univariable COX analysis. We further analysed the biological functions of these survival‐related SFs by functional enrichment analysis using the *WebGestalt* program (http://www.webgestalt.org/). The result suggested that cell cycle‐related genes (Figure 2C) and spliceosome structural proteins (Figure 2D) were over‐represented in the survival‐related SFs. We conducted a differential expression analysis of all 393 SFs and found that a small fraction (10 genes) were differentially expressed between normal and cancerous PCa tissues (Figure S1A–C). However, only one of these differentially expressed genes showed significance in the COX‐survival analysis. These results and previous reports led us to speculate that heterozygosity among tumour cells, rather than the general difference between tumour and normal tissue, may play a more important role in SF‐related tumour promotion effects.^25^

To explore the biological significance of the survival‐related SFs in large cohorts of PCa patients, we subjected these genes to *ConsensusClusterPlus* analysis in two independent PCa cohorts, TCGA and GSE21035. The results indicated that two distinct clusters are present in PCa patients based on the different gene expression patterns of the survival‐related genes (Figure 2E,F). Interestingly, these two distinct subtypes emerged with the highest stability (Figure 2E). The PCA analysis further suggested that the two subtypes of the PCa sample exhibit a clear difference in mRNA expression (Figure 2G,H). Notably, these two subtypes showed significant separation on the Kaplan–Meier plot, which suggests these SFs may play a significant role in the progression of PCa (Figure 2I,J).

### 3.3 The two SF subtypes have distinct pathway activation and immune modulation patterns

**FIGURE 3:**
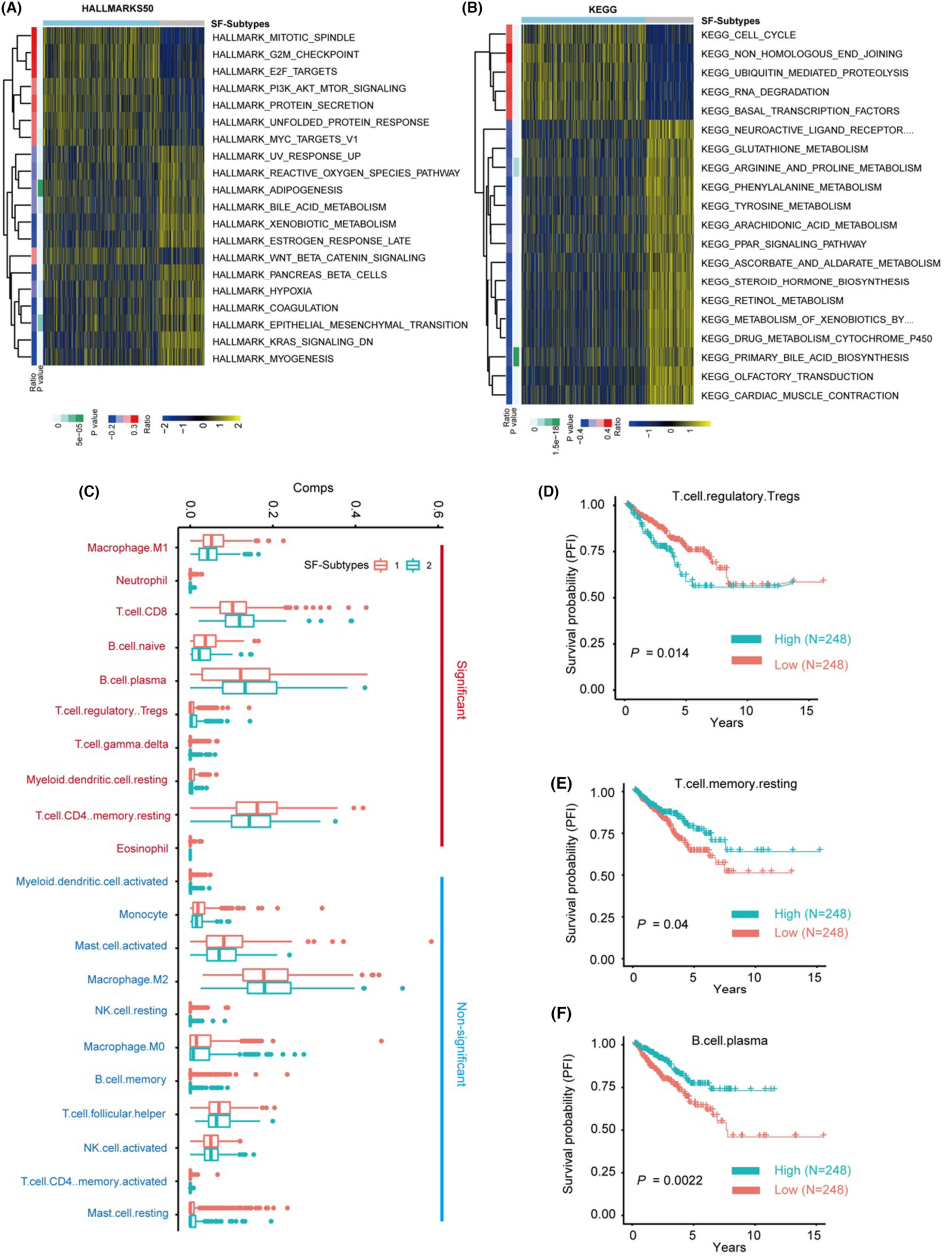
The two SF subtypes have distinct pathway activation and immune modulation patterns. (A, B) Heatmap showed the Gene Set Variation Analysis (GSVA) result of PCa samples in the TCGA‐PRAD dataset using the HALLMARK50 (A) and KEGG pathway (B) geneset. The result showed many cancer‐related pathways were differentially enriched in the two clusters. (C) Boxplot showed the levels of immune cell infiltration of PCa samples in the two clusters. The immune cell infiltration was analysed by the CIBERSORTx program using the LM22 dataset with default settings with 100 perturbations. (D–F) Kaplan–Meier analysis showed the infiltration levels of regulatory T cells (D), memory T cells (E) resting and plasma B cells (F) were significantly correlated to the PFI of PCa patients (log‐rank test).

Further, GSVA was carried out on the two PCa subtypes using the HALLMARKS50 gene set, and the results showed that some cancer‐promoting pathways, such as *oestrogen response*, *epithelial‐mesenchymal transition* and *KRAS signalling*, significantly enriched the subtypes with worse clinical outcomes (Figure 3A). In addition, GSVA using the KEGG gene set also showed enrichment of well‐known tumour‐associated metabolic‐related signalling pathways in the subtypes with worse clinical outcomes (Figure 3B), such as *arginine signalling*, indicating that dysregulation of SFs may trigger metabolic reprogramming and thus facilitate tumour progression. We further explored the infiltration patterns of immune cells in the two subtypes of PCa. We observed that 10 out of the 21 analysed immune cell types were differentially enriched in the two SFs‐based PCa subtypes (Figure 3C), including three prognosis‐related immune cell types: the regulatory T cells (Hazard, *p* = 0.014, Figure 3D), resting memory T cells (protective, *p* = 0.04, Figure 3E) and plasma B cells (protective, *p* = 0.002, Figure 3F). Collectively, these results suggest that intrinsic alterations of SFs exist in PCa, creating two distinct molecular subtypes with different molecular characteristics, prognosis indices and immune microenvironments. This indicates that the SF subtypes may be used as a supportive strategy to facilitate the diagnosis and prognosis of PCa.

### 3.4 The SF subtypes have significant differences in the general alternative splicing pattern

**FIGURE 4:**
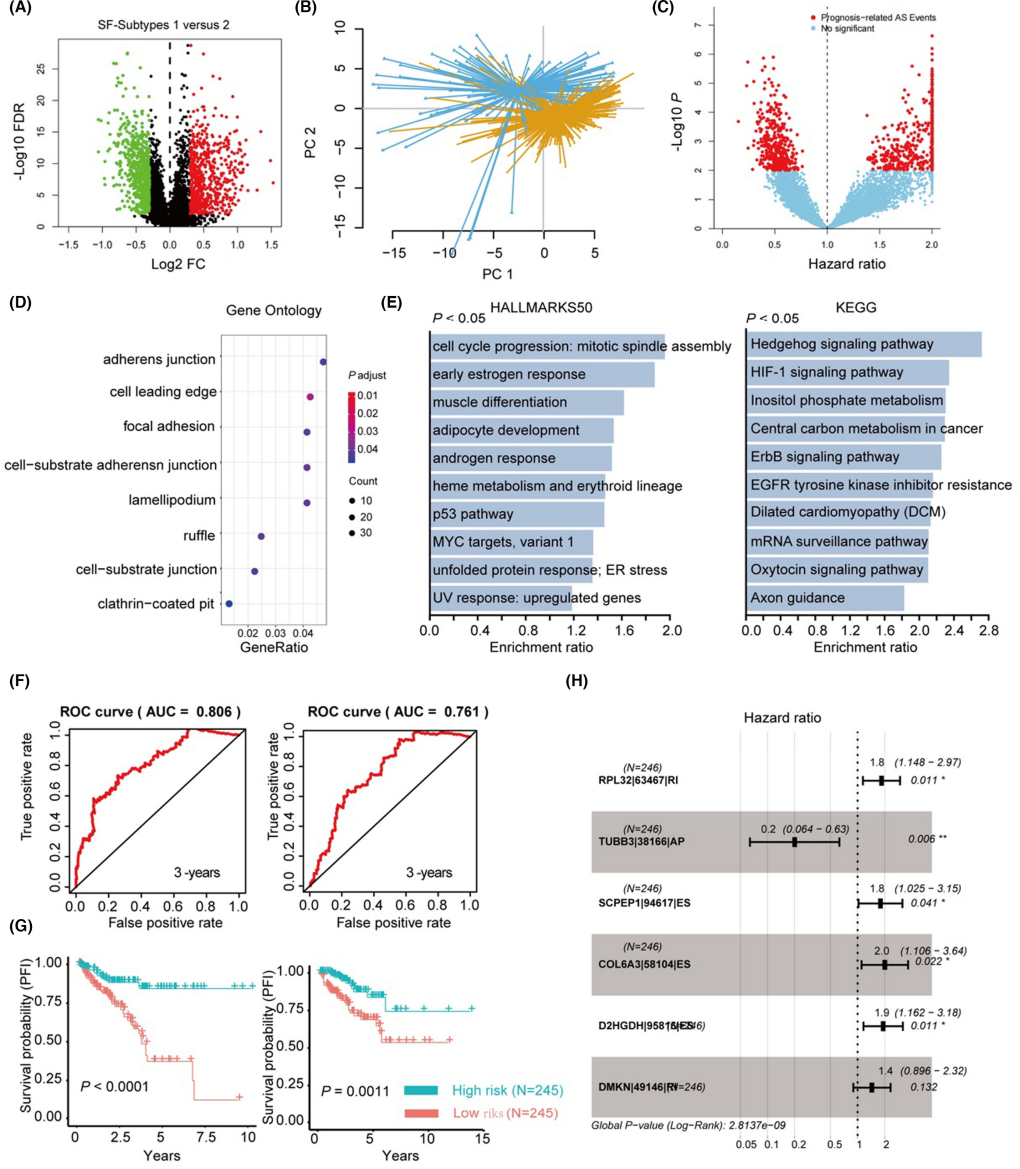
The identified two clusters of PCa patients have significant differences in the general alternative splicing pattern. (A) Volcano plots showed the differentially expressed alternative splicing (AS) events between the two identified SF subtypes. (B) PCA showed that the two SF subtypes have distinct expression patterns of AS‐events. (C) The volcano plot showed the distribution of hazard ratio and *p* values of all analysed AS events in TCGA‐PRAD dataset; the red dots indicated the survival‐significance AS‐events. (D) Overrepresentation analysis of the genes that related to survival‐significant AS events using Gene Ontology geneset. (E) Overrepresentation analysis of the genes that related to survival‐significant AS events using HALLMARKS50 and KEGG genesets. (F) Time‐dependent ROC curve analyses showed AUC values for the constructed AS‐risk‐models in the training dataset and testing dataset. (G) Kaplan–Meier plot showed the AS‐risk‐models can accurately predict the patient's BCR in the training and testing dataset (log‐rank test). (H) Forest plots showed the multivariable Cox regression analysis of key genes in SF‐risk‐models.

The SF subtypes show significant differences in the general pattern of alternative splicing. As the ConsensusCluster result predicted two consensus PCa subtypes, and considering that alternative splicing events are controlled and influenced by SFs, we next examined the pattern of alternative splicing in the two identified SF subtypes. Differential expression analysis suggested that 1251 out of 7704 (16.2%) AS events were differentially expressed in the two PCa subtypes (Figure 4A). This result was consistent with the PCA results, which showed that the overall pattern of AS events was distinct in the two SF subtypes (Figure 4B). Univariable‐COX analysis identified 298 survival‐related AS events that were subjected to enrichment analysis (Figure 4C, Table S2). GO enrichment analysis showed that signalling pathways such as *adhering junction*, *cell leading edge*, *focal adhesion* and *cell‐substrate junctions* were significantly enriched. This suggests that the dysregulated AS events may be linked with the metastatic potential or/and mobility of PCa cells (Figure 4D). Meanwhile, HALLMARKS50 and KEGG enrichment analysis showed that *cell cycle progression*, *MYC targets* and *HIF‐α signalling pathway* may also be controlled by these AS events (Figure 4E).

Furthermore, we evaluated the clinical significance of the identified 57 AS events that were both dysregulated in the two SF subtypes and significantly associated with PFI. COX‐lasso regression analysis was used to construct a survival prediction model. The results showed that a risk prediction model with promising survival predicting power was successfully constructed. ROC analysis (Figure 4F) followed by Kaplan–Meier analysis (Figure 4G) showed that the constructed risk prediction model has good predictive power for the PFI of PCa, with AUC >0.750 in both the training and testing datasets. The multivariable COX analysis also identified six survival‐associated AS events with a *p* value <0.05, and the detailed results are shown in Figure 4H. These data suggest that the identified two SF subtypes of PCa patients have significant differences in the general alternative splicing pattern and the identified key AS events may be linked with the prognosis and diagnosis of PCa.

### 3.5 Construction of survival prediction classifier using the survival‐related SFs

**FIGURE 5:**
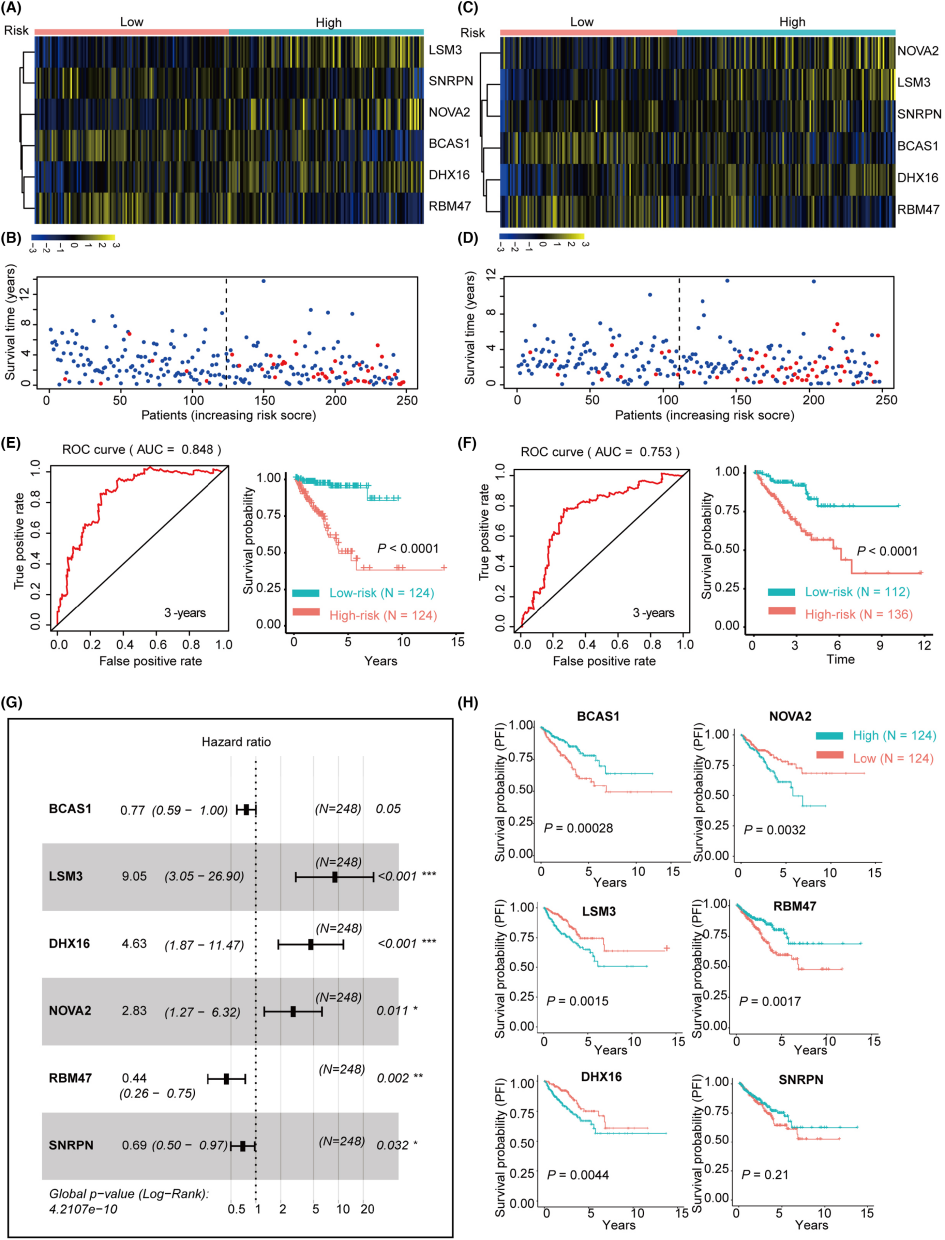
Construction of survival prediction classifier using the survival‐related SFs. (A) Heatmap showed the expression patterns of the six key genes of high‐ and low‐risk groups in the training dataset. (B) Dot plot showed the association between the classifier‐calculated RiskScores and BCR in the training datasets. (C) Heatmap showed the expression patterns of the six key genes of high‐ and low‐risk groups in the testing dataset. (D) Dot plot showed the association between the classifier‐calculated RiskScores and BCR in the testing datasets. (E, F) Time‐dependent ROC analysis and Kaplan–Meier analysis (log‐rank test) indicated the SF‐classifier has promising prediction power of PFI in the training (E) and testing (F) dataset. (G) Forest plot showing the multivariable Cox regression analysis of six key genes used in the SF‐classifier. (H) Kaplan–Meier analysis of the six key genes (BCAS1, NOVA2, LSM3, RBM47, DHX16, SNRPN) showed that both of them were significantly associated with PFI in TCGA‐PRAD dataset (log‐rank test).

As the bioinformatics analysis showed that dysregulation of specific SFs was linked with the progression of PCa, a survival prediction classifier was constructed using the LASSO‐COX method, as previously described. Initially, the survival‐related SFs with a COX *p* < 0.01 were subjected to LASSO regression followed by multivariable COX regression analysis to calculate the coefficients using the TCGA‐PRAD training dataset (Table S2). Then, a survival prediction classifier was constructed, and the RiskScore of each patient was calculated. Results showed that this six‐gene risk prediction classifier (SF‐classifier) can stratify PCa patients into high‐ and low‐risk groups with a significant difference in clinical outcome in both the training (Figure 5A,B) and testing sets (Figure 5C,D). ROC analysis and Kaplan–Meier analysis also showed good predictive power of this model with an AUC >0.7 in both the training (Figure 5E) and testing datasets (Figure 5F). Furthermore, the multivariable COX analysis showed that the six key SFs were independent factors that correlated with the PFI of PCa patients (Figure 5G). Additionally, Kaplan–Meier analysis showed that all six genes were significantly associated with the PFI of PCa patients (Figure 5H). The expression of the six key SFs and the calculated RiskScores are shown in Table S3.

### 3.6 Verification of the SF‐classifier in three independent external datasets

**FIGURE 6:**
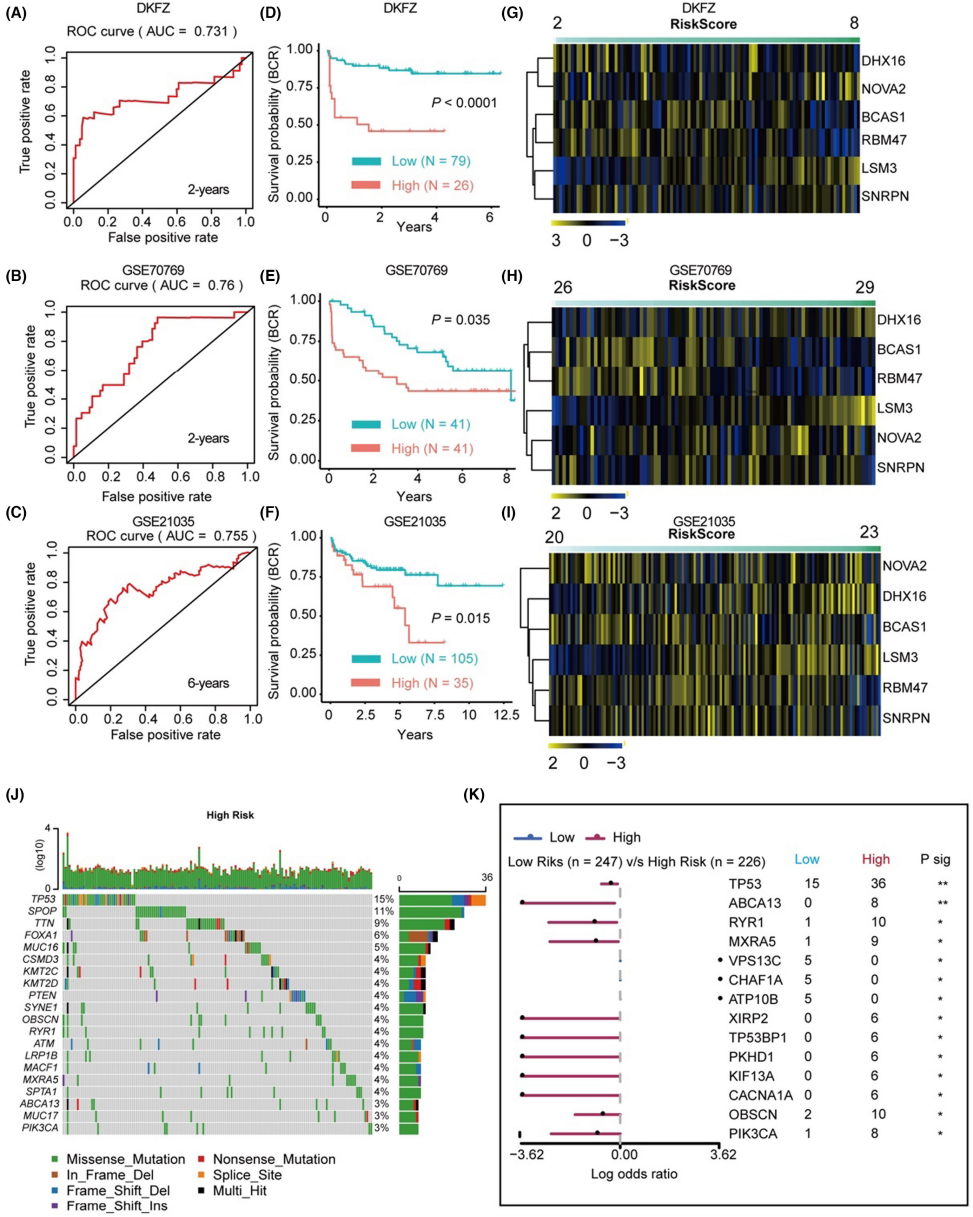
Verification of the SF‐classifier using three independent external datasets. (A–C) Time‐dependent ROC analysis of the SF‐classifier in DKFZ‐dataset (A), GSE70769 dataset (B) and GSE21035 dataset (C). (D–F) Kaplan–Meier analysis (by log‐rank test) of the SF‐classifier in DKFZ‐dataset (D), GSE70769 dataset (E) and GSE21035 dataset (F). (G–I) Heatmap showed the expression pattern of six‐key genes and SF classifier‐calculated RiskScores in DKFZ‐dataset (G), GSE70769 dataset (H) and GSE21035 dataset (I). (J) Waterfall plot showed the top‐ranked somatic mutation pattern of high‐ and low‐risk groups predicted by the SF‐classifier in TCGA‐PRAD dataset. (K) The forest plot showed the statistical information of the top‐ranked somatic mutation events in the high‐ and low‐risk groups.

Next, we verified the predictive power of the classifier in three external independent PCa cohorts, DKFZ, GSE70769 and GSE21035, respectively. ROC analysis (Figure 6A–C) and Kaplan–Meier survival analysis (Figure 6D–F) both suggest promising predictive power of the constructed classifier (*p* < 0.05 and AUC > 0.700 for all datasets). In addition, the associations between RiskScores and the expression patterns of key SFs in the DKFZ, GSE70769 and GSE21035 datasets are shown in Figure 6G–I, respectively; the details are provided in Table S3. Additionally, we analysed the mutation spectrum of the high‐ and low‐risk patients predicted by the classifier. The results indicated that many key genes were more frequently mutated in the high‐risk group compared to the low‐risk group, such as TP53 (Tumour Protein P53), SPOP (Speckle‐Type POZ Protein), FOXA1 (Forkhead Box A1) and so forth (Figure 6J). Interestingly, TP53 showed the highest mutation rate in the high‐risk group, which suggests that dysregulation of the six identified SFs may contribute to the gain of mutation of TP53 in PCa (Figure 6K). The expression of the six key SFs and the calculated RiskScores are shown in Table S3.

### 3.7 Comparison of the prognosis prediction power between SF‐classifier and other clinicopathologic parameters

**FIGURE 7:**
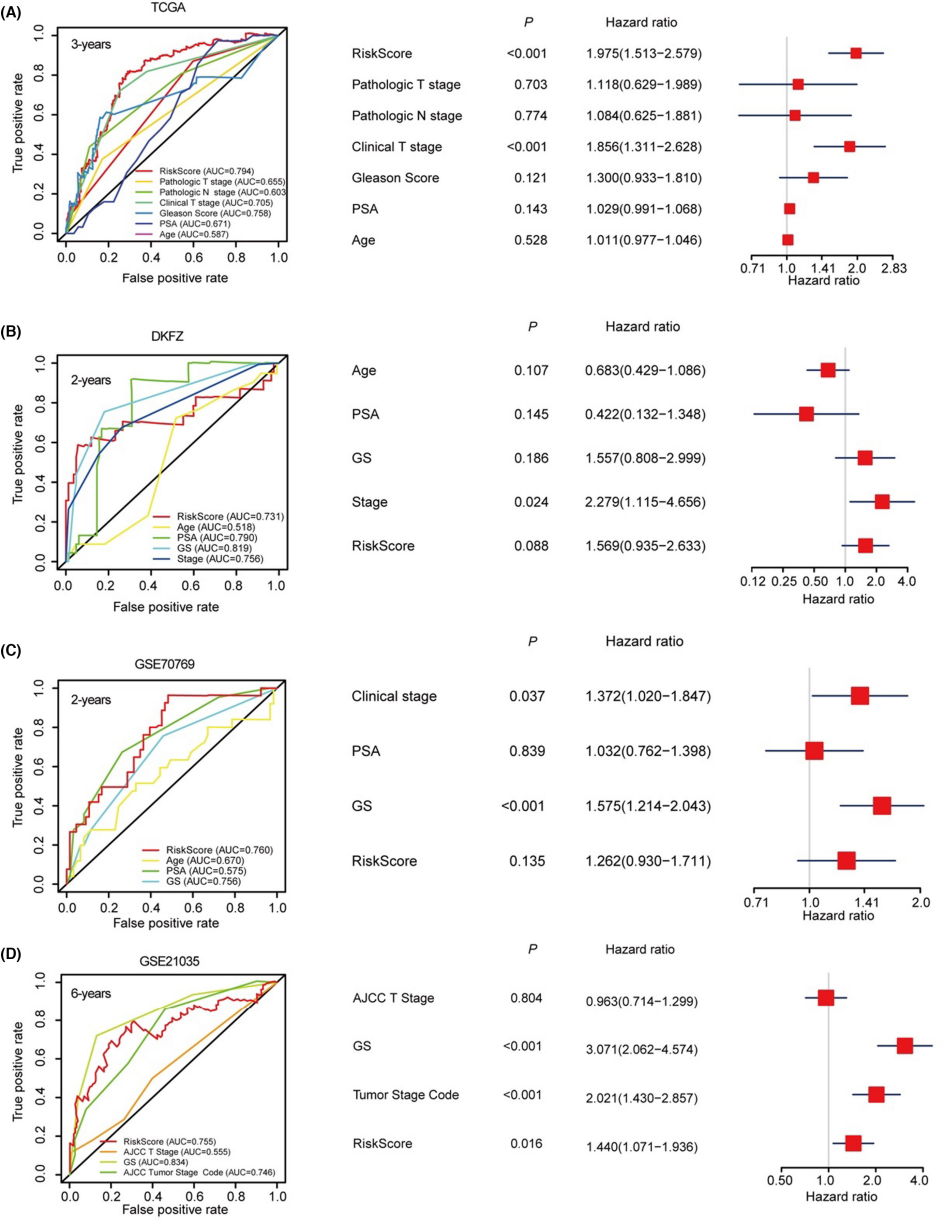
Comparison of the prognosis prediction power between SF‐classifier and other clinicopathologic parameters. (A) Time‐dependent ROC and multivariable‐COX analyses of the BCR survival‐predicating capabilities of RiskScore and other clinicopathologic parameters (pathological T stage, pathologic N stage, clinical T stage, clinical N stage, Gleason score, PSA and Age) in TCGA‐PRAD dataset. (B) Time‐dependent ROC and multivariable‐COX analyses of the BCR survival‐predicating capabilities of RiskScore and other clinicopathologic parameters (Age, PSA, Gleason score, stage) in DKFZ (Deutsches Krebsforschungszentrum) dataset. (C) Time‐dependent ROC and multivariable‐COX analyses of the BCR survival‐predicating capabilities of RiskScore and other clinicopathologic parameters (clinical stage, PSA and Gleason score) in GSE70769 dataset. (D) Time‐dependent ROC and multivariable‐COX analyses of the BCR survival‐predicating capabilities of RiskScore and other clinicopathologic parameters (AJCC T stage, Gleason score and tumour stage code) in GSE21035 (SMKCC) dataset.

We also examined the prognosis prediction power of the SF‐classifier in comparison with other well‐established clinicopathological parameters, including PSA value, Gleason Score, tumour stage and age, according to the available information in each dataset. In the TCGA cohort, the RiskScore showed the highest AUC value and a significant multivariable‐COX hazard ratio (Figure 7A). In the DKFZ, GSE70769 and GSE21035 datasets, RiskScores showed very promising prediction power with AUC >0.700, but they exhibited less robustness compared with some other parameters, especially the Gleason score (Figure 7B–D). Although multivariable‐COX analysis indicated that the calculated RiskScores were independent prognosis factors in the TCGA (Figure 7A) and GSE21035 (Figure 7D) datasets with a *p* value less than 0.05, results from DKFZ (Figure 7B) and GSE70769 (Figure 7C) suggested that the RiskScore should be combined with other clinicopathological parameters such as Gleason score (GS) and Stage to accurately predict BCR in PCa patients. Collectively, these analyses suggested that a six‐SF‐based classifier was successfully constructed with promising prognosis prediction power in PCa patients.

### 3.8 Key SFs are involved in the progression of PCa

**FIGURE 8:**
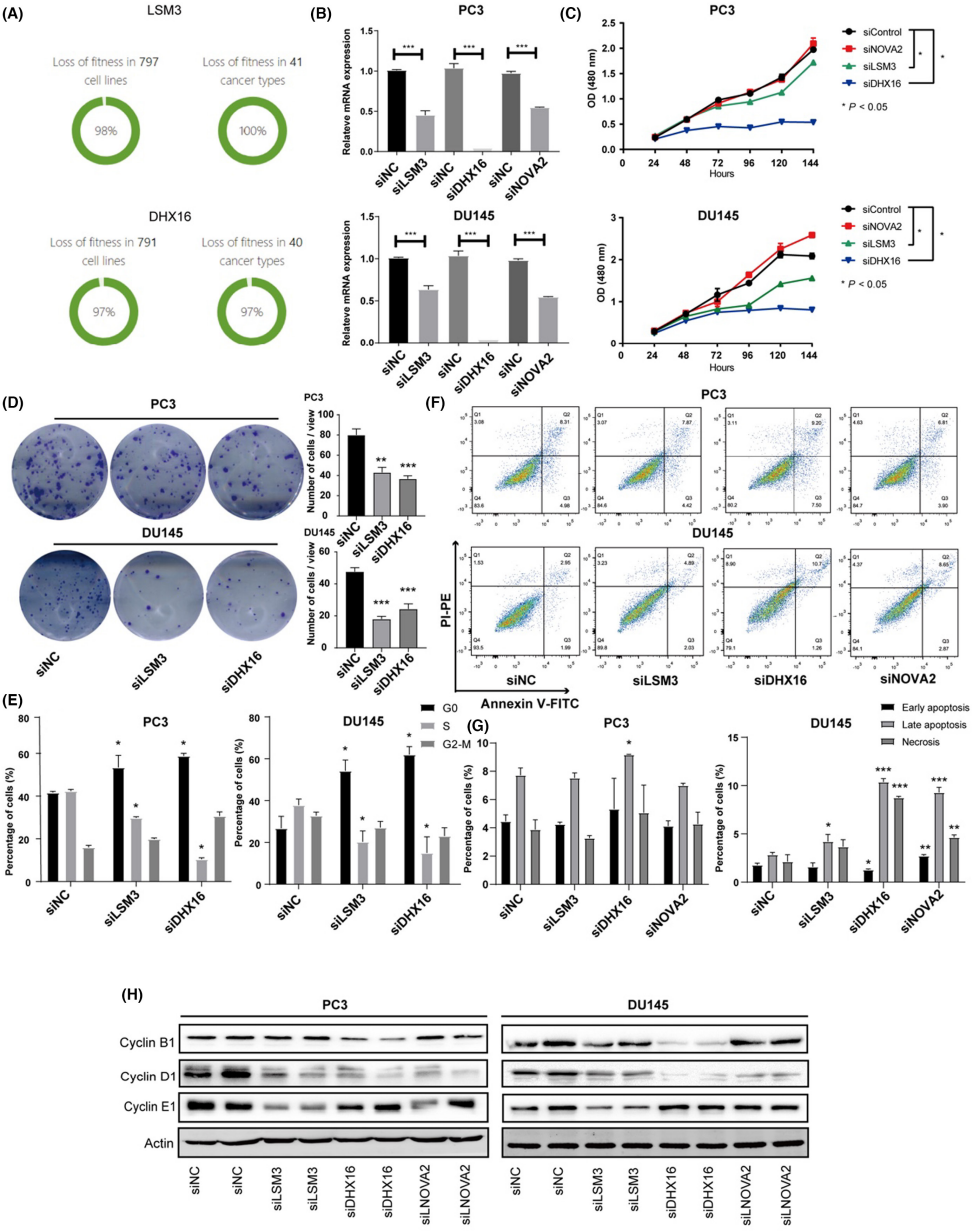
Key SFs are involved in the progression of PCa. (A) CRISPR‐CAS9 screening data (analysed by the Project Score database) showed LSM3 and DHX16 are fitness‐essential genes across a variety of cell lines and cancer types. (B) Quantitative real‐time PCR results showed the efficacy of siRNA‐induced gene silencing of LSM3, DHX16 and NOVA2 (Student's *t*‐test). (C) Cell counting kit 8 (CCK‐8) assay showed that silencing of LSM3 and DHX16 significantly inhibits the proliferation of two PCa cell lines, PC3 and DU145 (anova‐test). (D) Colony formation assay indicated that the knockdown of LSM3 and DHX16 significantly inhibit the proliferation of two PCa cell lines (Student's *t*‐test). (E) Follow cytometry showed that silencing of LSM3 and DHX16 inhibits the cell cycle progression of PCa cell lines (Student's *t*‐test). (F, G) Cell apoptosis was significantly promoted upon silencing of LSM3, DHX16 and NOVA2, as analysed by Follow cytometry assay in PC3 and DU145 cells (Student's *t*‐test). (H) Western blot analysis of key genes involved in cell‐cycle regulation in PC3 cells that silenced with LSM3, DHX16 or NOVA2.

The above in silico analyses have demonstrated that dysregulation of the six SFs is associated with poor clinical outcomes, as well as the progression of PCa. Next, we aimed to understand the biological significance of the six identified key SFs in PCa. We first analysed the high‐throughput CRISPR screening data using the Project Score database (https://score.depmap.sanger.ac.uk/). Notably, the results showed that LSM3 and DHX16 were significantly associated with the fitness of prostate cancer cells, suggesting that these two SFs might be involved in controlling key processes for tumour cell survival and proliferation (Figure 8A).

To further assess the biological significance of NOVA2, LSM3 and DHX16 in PCa cells, in vitro functional studies were carried out. The siRNAs that specifically targeted NOVA2, LSM3 and DHX16 were designed and transfected into PC3 and DU145 cells (Figure 8B). We found that the knockdown of LSM3 and DHX16, but not NOVA2, significantly inhibited the proliferation of two PCa cell lines, PC3 and DU145, as revealed by the CCK8 assay (Figure 8C) and the colony formation assay (Figure 8D). Consistently, we observed that silencing LSM3 and DHX16 altered the cell cycle progression of PCa cells (Figures 6E and S2). Interestingly, although the knockdown of NOVA2 had no significant influence on cell proliferation, we found that down‐regulation of both LSM3, DHX16 and NOVA2 induced cell apoptosis in DU145 cells (Figure 8F,G). However, NOVA2 did not affect the apoptosis of PC3 cells, and LSM3 did not associate with the apoptosis in PC3 cells (Figure 8F,G). Furthermore, western blot analyses were carried out to examine the key proteins that regulated cell cycle progression upon silencing LSM3 and DHX16. The results showed that the protein levels of Cyclin B1, Cyclin D1 and Cyclin E1 were influenced by the knockdown of LSM3 and DHX16 (Figure 8H). Collectively, these results strongly indicated that LSM3 and DHX16 were involved in controlling key checkpoints in cell cycle progression, and the function of NOVA2 and LSM3 may be context‐dependent.

## 4 DISCUSSION

Tumour progression is a multi‐step process that involves intricate regulatory mechanisms. Emerging evidence suggests that alternative splicing (AS) of mRNA plays a crucial role in this process. Gene expression is a sophisticated cascade where AS is instrumental; it is believed that approximately 90% of mRNAs in eukaryotic cells are regulated by this mechanism. This confers an additional layer of gene to protein regulation, extending the traditional boundaries of the well‐established ‘central dogma’.^4^, ^6^, ^26^ SFs are a class of proteins that regulate the alternative splicing of mRNAs, and their aberrant expression is involved in various aspects of tumour progression, such as cell cycle regulation, epithelial‐mesenchymal transition in tumours, cell stemness and immune escape of tumours.^6^ Therefore, systematic analysis of SFs abnormalities is important for gaining insight into the mechanisms that drive tumour progression.

Several studies have reported the role of SFs in PCa. Labrecque MP et al. found that SRRM3 and SRRM4 were associated with PCa castration‐resistant neuroendocrine PCa.^13^ Kawamura N et al. found that SF3B2 could promote PCa progression by regulating isoform switching of the mRNA encoding androgen receptor.^27^ However, considering the possible complex expression patterns of SFs in PCa, there is a limitation in relevant studies to systematically and comprehensively analyse the role of SFs in PCa. In the present study, we first analysed 393 SFs in prostate cancer through univariable COX analysis and differential expression analysis. We found that about 15% of these SFs correlated with prognosis, suggesting their potential involvement in PCa progression. Furthermore, using ConsensusClusterPlus analysis, we identified 59 survival‐related SFs that stratified PCa patients into two distinct molecular subtypes with different clinical outcomes. These data indicate the existence of two specific expression patterns of SFs in PCa, resulting in two distinct clusters of gene expression profiles, signalling pathways and AS events. This point is further supported by PCA, GSVA and differential expression analysis of AS events. Of interest, many metabolism‐related signalling pathways, such as tyrosine metabolism and glutathione metabolism, were significantly activated in the molecular subtype with a poorer prognosis, suggesting that the abnormalities of SFs may lead to complex metabolic reprogramming in PCa cells, which in turn drive tumour progression. On the contrary, we found that survival‐related SFs were significantly associated with the cell cycle. Furthermore, AS events that were differentially expressed in the two SF subtypes could also be significantly enriched in cell cycle‐related pathways, suggesting that the dysregulated expression of SFs may regulate cell cycle‐related AS events and ultimately lead to the accelerated progression of the cell cycle in PCa cells. Consistent with this, the cell function experiments demonstrated that the knockdown of two key SFs, LSM3 and DHX16, significantly resulted in the blockage of cell cycle progression and thus led to the inhibition of proliferation in PC3 and DU145 cells.

The involvement of splicing factors in the progression of prostate cancer and other types of cancers has been highlighted in several studies. For instance, RNA splicing factors have been found to promote the expression of the androgen receptor and its variants, which is crucial for the progression of prostate cancer. This implies that splicing factors might be potential therapeutic targets. From a diagnostic perspective, the SFs we identified could potentially serve as novel biomarkers for PCa. The SF‐based classifier we established demonstrated a promising prognosis‐prediction performance, suggesting that these SFs might be used in a clinical setting to help stratify patients based on their predicted disease outcomes. This could aid in making more informed decisions about the aggressiveness of the therapeutic approach.

In this work, we constructed an SF classifier using the survival‐significant SFs, and six key SFs were identified. Three of them, BCAS1, RBM47 and SNRPN, are biomarkers for a good prognosis in PCa patients. To date, little is known about the association of BCAS1 with PCa. BCAS1 has been found to be highly expressed in breast cancer cell lines.^28^ Consistent with our results, Shuqiang Li reported that it can serve as a biomarker to predict castration‐resistant PCa.^29^ RBM47 has been shown to inhibit the progression of several malignancies, including thyroid carcinoma, nasopharyngeal carcinoma, lung cancer and colorectal cancer, whereas its role in PCa has not been characterized.^30^, ^31^, ^32^, ^33^ Consistent with these reports, we found that RBM47 is a favourable prognostic marker in PCa. Further experimental studies are needed to illustrate its specific mechanism in the progression of PCa. SNRPN is a key component of the small nuclear ribonucleoprotein complex, which functions in pre‐mRNA processing and may contribute to tissue‐specific alternative splicing. However, its functional roles in cancer remain obscure. We found that in PCa, SNRPN showed significance associated with prognosis in the multivariate COX analysis, where its high expression indicated a favourable prognosis.

NOVA2 has been identified to play important roles in a variety of biological processes, such as circRNA biogenesis, neurodevelopmental phenotypes, as well as cancer.^34^, ^35^, ^36^, ^37^ However, no study has reported the association between NOVA2 and PCa. In this study, we found that high expression of NOVA2 was associated with a worse prognosis in patients with PCa. Unfortunately, the CRISPR/CAS9 screening data, as well as the cell‐level functional analyses, showed that NOVA2 was not associated with the proliferation and cell cycle of PCa cells. As for DHX16, it has been suggested to be involved in multiple biological processes, such as innate antiviral immunity and pre‐mRNA splicing, but it has not received attention in the field of cancer.^38^, ^39^ We found its high expression significantly associated with a worse prognosis of PCa patients. Notably, both the CRISPR/CAS9 screening data and our in vitro experiment indicated that it played important roles in facilitating the proliferation and survival of PCa cells. The LSM family of proteins has been found to have potential therapeutic and prognostic value in breast cancer.^40^ We have previously reported that LSM3 can serve as a subtype‐specific biomarker in basal‐like breast cancer.^5^ Here, we found that its high expression strongly associates with a shorter biochemical recurrence (BCR) of PCa patients with the highest hazard ratio among the six identified key genes in the SF‐classifier. More importantly, the knockdown of LSM3 significantly inhibits the proliferation of PCa cells. Further molecular biology investigations are suggested to elucidate LSM3 and DHX16's mechanisms in prostate cancer progression. Functional assays, including migration and invasion studies, can be conducted following LSM3 and DHX16 manipulation to assess their influence on cellular behaviour. Co‐immunoprecipitation can identify potential interacting partners, revealing their roles in broader pathways. Gene expression profiling post LSM3/DHX16 modulation could expose downstream targets and implicated pathways. Additionally, in vivo studies using appropriate models could inform about these genes' role in tumour growth and metastasis. Larger, more diverse clinicopathological studies can then contextualize these mechanistic insights to specific patient cohorts. Through such comprehensive approaches, the roles of LSM3 and DHX16 in prostate cancer progression can be better understood.

In summary, we constructed an SF classifier based on survival‐significant SFs, which have promising prognosis prediction power across four independent datasets and showed comparable accuracy with other well‐established parameters such as PSA value and Gleason score. Although some similar studies have established several prognosis models using other gene sets, to our best knowledge, no study has systematically analysed the prognosis values of SFs in PCa. Here, we identified six key SFs as new multidirectional biomarkers for PCa that may also serve as targets for the treatment and intervention of PCa. Acknowledging the limitations of our study, it is evident that the SF classifier, although insightful, did not distinctly outperform other established prognosis parameters, indicating the need for further optimization and verification using more diverse and extensive data sets. In particular, our research employed four PCa cohorts that predominantly consisted of Caucasian individuals, and therefore, may not capture the complete gene expression spectrum representative of diverse PCa populations. For example, populations like the Chinese, with unique genetic backgrounds, may exhibit different disease susceptibility and progression. These limitations underscore the importance of future research utilizing local and diverse cohorts to ensure a comprehensive understanding of PCa. Furthermore, while our study identified potential roles for LSM3 and DHX16 in PCa progression, the mechanistic pathways remain unclear, necessitating more in‐depth molecular biology investigations.

## AUTHOR CONTRIBUTIONS

**He zhang:** Conceptualization (equal); data curation (equal); formal analysis (equal); funding acquisition (equal); investigation (equal); methodology (equal); writing – original draft (equal); writing – review and editing (equal). **Jianfei Tian:** Data curation (equal); formal analysis (equal). **Sixin Ren:** Data curation (equal); formal analysis (equal); investigation (equal). **Baoai Han:** Data curation (equal); formal analysis (equal); investigation (equal); methodology (equal). **Ruinan Tian:** Data curation (equal); formal analysis (equal); investigation (equal). **Xiaoyan Zuo:** Data curation (equal); formal analysis (equal); investigation (equal). **Hui Liu:** Data curation (equal); investigation (equal). **Zhiyong Wang:** Formal analysis (equal); investigation (equal). **Yanfen Cui:** Data curation (equal); funding acquisition (equal); investigation (equal). **Liming Liu:** Formal analysis (equal); funding acquisition (equal); investigation (equal); writing – original draft (equal); writing – review and editing (equal). **Hui Guo:** Formal analysis (equal); investigation (equal). **Fei Zhang:** Conceptualization (equal); data curation (equal); formal analysis (equal); funding acquisition (equal); investigation (equal); methodology (equal); project administration (equal); resources (equal); supervision (equal); validation (equal); visualization (equal); writing – original draft (equal); writing – review and editing (equal). **Ruifang Niu:** Conceptualization (equal); data curation (equal); formal analysis (equal); funding acquisition (equal); investigation (equal); methodology (equal); project administration (equal); supervision (equal); validation (equal); visualization (equal); writing – original draft (equal); writing – review and editing (equal).

## FUNDING INFORMATION

This work was supported by grants from the National Natural Science Foundation of China (Numbers 82073252, 81903092, 82073085, 82203687 and 32000443), Tianjin Municipal Science and Technology Commission (Number 20JCZDJC00030), and Changjiang Scholars and Innovative Research Team (Number IRT_14R40).

## CONFLICT OF INTEREST STATEMENT

The authors declare no potential conflict of interest.

## Supporting information


Figure S1.

Figure S2.

Table S1.

Table S2.

Table S3.

Data S1.

## Data Availability

All datasets used in this study are publicly available from the corresponding database or provided as Supplementary Materials.
