# Correction to “Comprehensively Analysis of Splicing Factors to Construct Prognosis Prediction Classifier in Prostate Cancer”

**DOI:** 10.1111/jcmm.70743

**Published:** 2025-07-30

H. Zhang, J. Tian, S. Ren, B. Han, R. Tian, X. Zuo, H. Liu, Z. Wang, Y. Cui, L. Liu, H. Guo, F. Zhang, and R. Niu, “Comprehensively Analysis of Splicing Factors to Construct Prognosis Prediction Classifier in Prostate Cancer,” *Journal of Cellular and Molecular Medicine* 27, no. 18 (2023): 2684–2700, https://doi.org/10.1111/jcmm.17849.

Concerns were raised by a third party regarding the shape of the ROC curves (Figures 4–6) and the apoptosis measurements in Figure 8 of this article.

As stated by the authors, the observed irregularity in the curve shape results from methodological distinctions between the Kaplan–Meier (KM) and nearest neighbour estimation (NNE) approaches in the ‘survivalROC’ analysis package (R software, version 4.2.1). The selection of KM methodology was based on three considerations: (1) The results of NNE are highly dependent on the choice of span parameter; (2) KM provides reproducible results without parameter tuning requirements; and (3) KM maintains data fidelity despite producing less visually smooth curves. This methodological decision prioritises analytical precision over aesthetic considerations.

Regarding the apoptosis measurements, the main concern was that based on the data presented and the apoptosis detection kit used, it is not possible to clearly distinguish between late apoptotic and necrotic cells. The fraction of cells labelled as ‘necrotic’ in Figure 8G could be an artifact due to mechanical damage of the cell membrane and false positive cytoplasmic RNA staining by PI. Additional technical issues were identified regarding the lack of manual compensation of the FACS samples in Figure 8F, leading to incorrect live, early apoptotic, and late apoptotic cell population percentages; therefore, incorrect FACS analysis results in Figure 8F,G. Hence, the authors were asked to repeat the experiments in question to provide further clarification.

The new results confirmed the conclusions presented in section 3.8 of the published manuscript: ‘down‐regulation of both LSM3, DHX16 and NOVA2 induced cell apoptosis in DU145 cells (Figure 8F,G)’ ‘NOVA2 did not affect the apoptosis of PC3 cells, and LSM3 did not associate with the apoptosis in PC3 cells (Figure 8F,G)’, the experimental results and conclusions of the paper remain unaffected.

The corrected Figure 8F,G are as follows:

**FIGURE 8:**
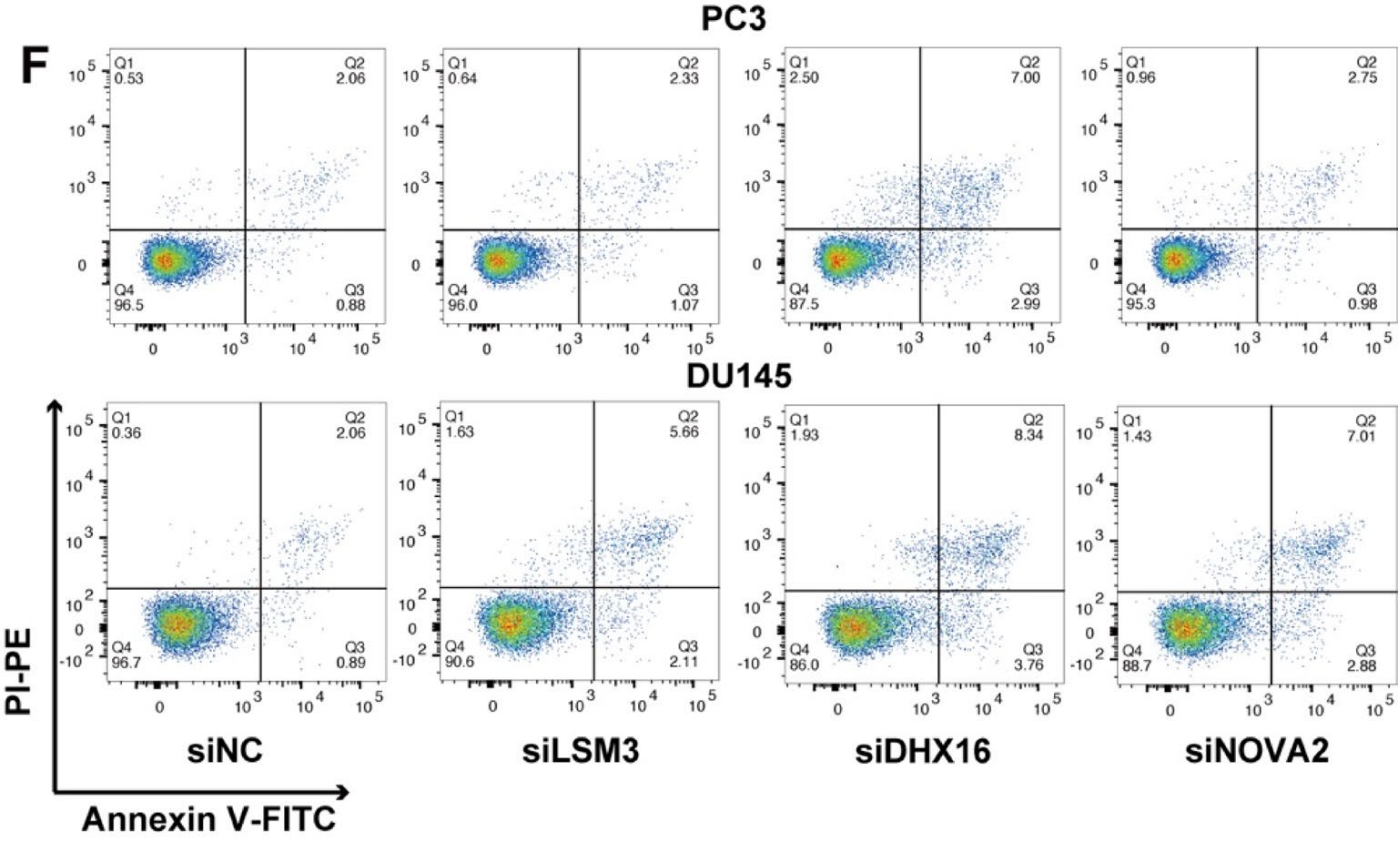
**(F)** Representative FACS profiles showed that cell apoptosis was significantly promoted upon silencing of LSM3, DHX16, and NOVA2 in PC3 and DU145 cells.

**FIGURE 8:**
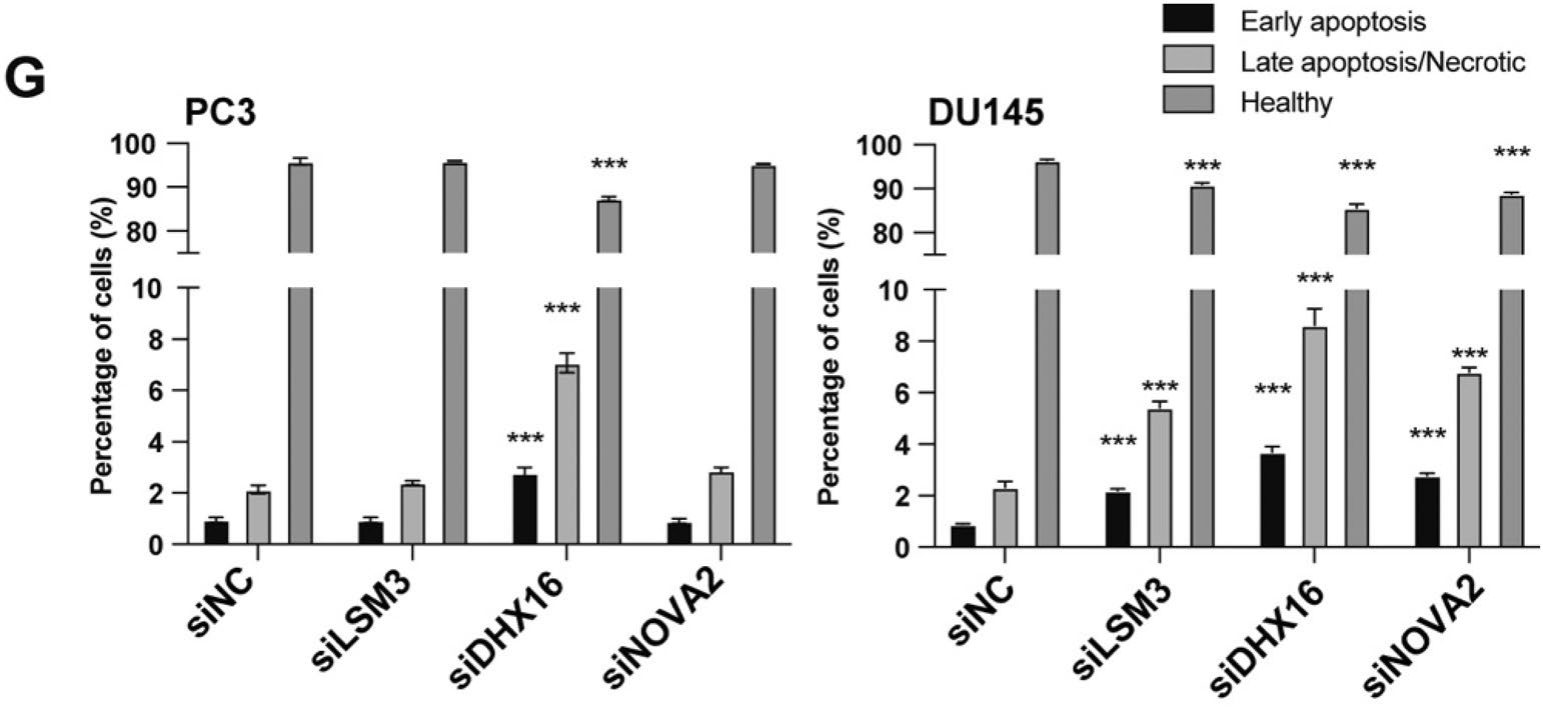
**(G)** Barplots showed the proportions of healthy (Q4: Annexin V− and PI−), early apoptosis (Q3: Annexin V+ and PI−) and late apoptosis/necrotic (Q2: Annexin V+ and PI+) cells in the FACS experiments (Student's *t*‐test).

