# Supplementary figures and images for: Comprehensively analysis of splicing factors to construct prognosis prediction classifier in prostate cancer
Source: J Cell Mol Med. 2023 Aug 9;27(18):2684–700. doi: 10.1111/jcmm.17849 (PMC10494302; doi:10.1111/jcmm.17849)

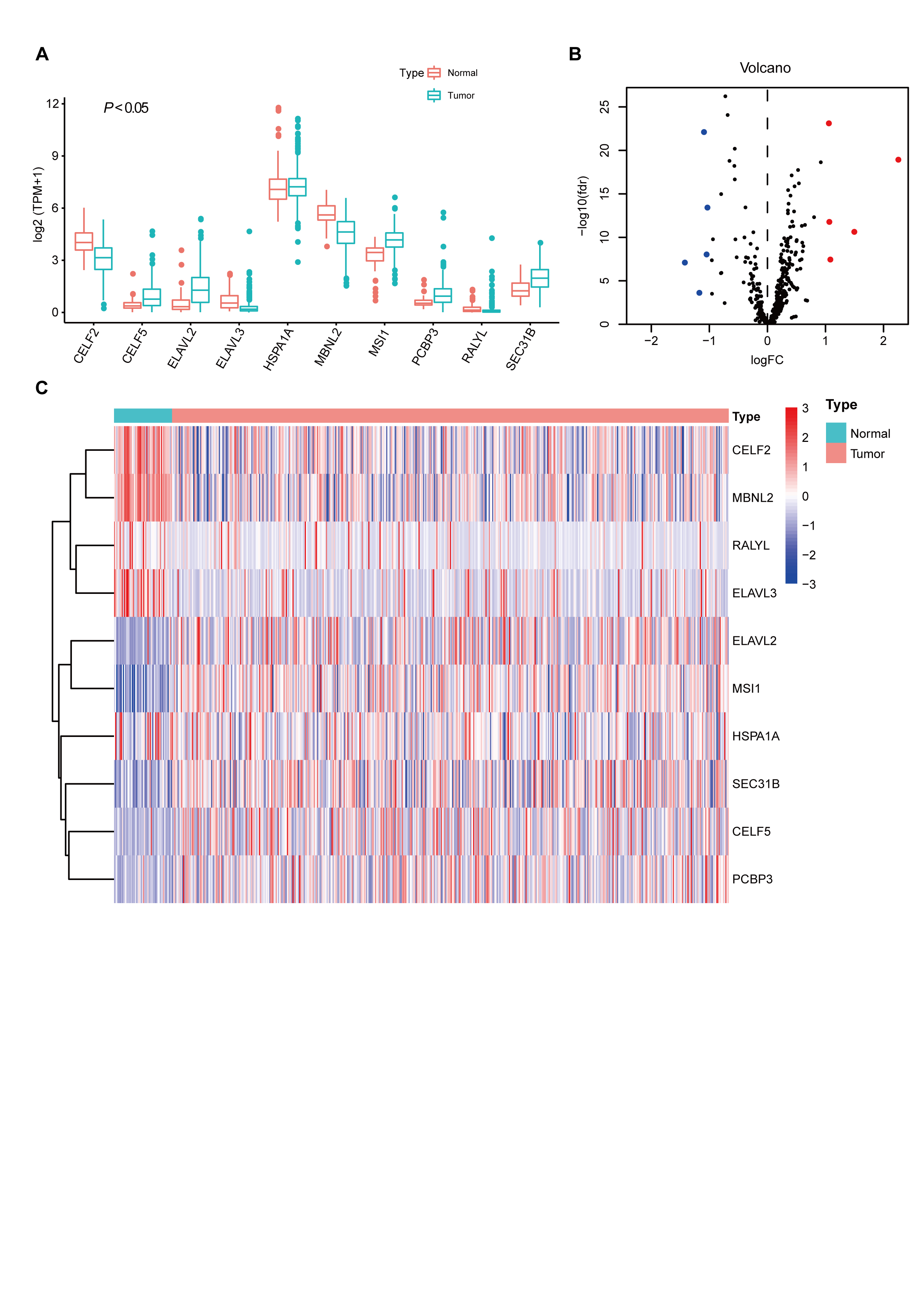

Supplement: Supplementary file 1 — Figure S1. [file JCMM-27-2684-s006.jpg]

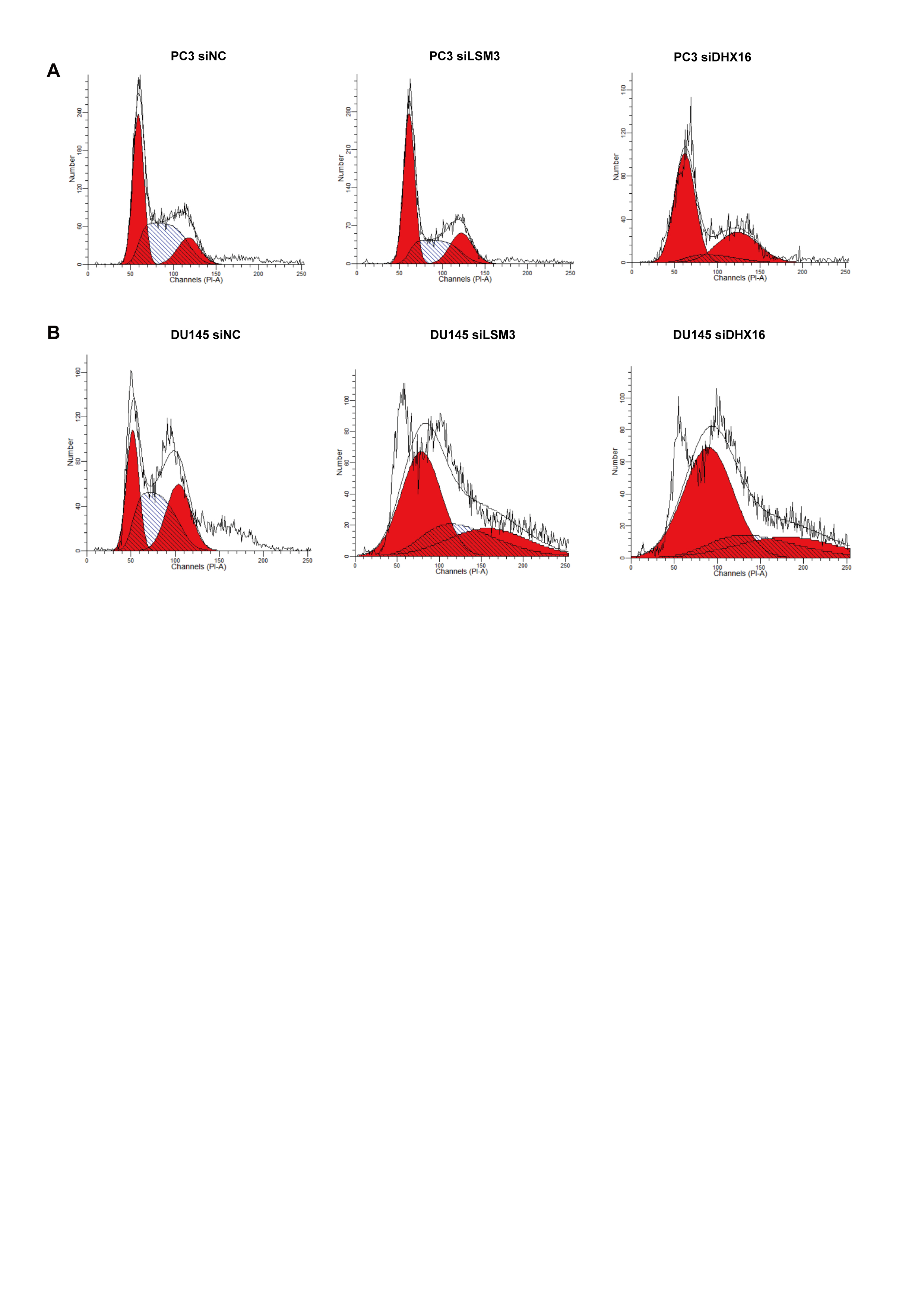

Supplement: Supplementary file 2 — Figure S2. [file JCMM-27-2684-s001.jpg]
