# Supplementary material for: Comprehensively analysis of splicing factors to construct prognosis prediction classifier in prostate cancer
Source: J Cell Mol Med. 2023 Aug 9;27(18):2684–700. doi: 10.1111/jcmm.17849 (PMC10494302; doi:10.1111/jcmm.17849)
Supplement: Supplementary file 6 — Data S1. [file JCMM-27-2684-s005.docx]

**Supplementary Figure Legends**

**Supplementary Figure S1. The differentially expressed splicing factors in the TCGA-PRAD dataset**

**(A)** Barplot showed the expression distribution of differentially expressed splicing factors (SFs) among normal and cancerous tissues in TCGA-PRAD dataset. **(B)** Volcano plot showed the *P* value and fold-change (logFC) of SFs in TCGA-PRAD dataset. **(C)** Heatmap showed the expression pattern of differential SFs across all TCGA-PRAD samples.

**Supplementary Figure S2. Cell cycle progression was altered in LSM3 and DHX16 silenced PCa cells.**

**(A, B)** The cell cycle was analyzed by flow cytometry in two PCa cells, PC3 **(A)** and DU145 **(B)**, respectively. The cells were transfected with siRNA that targeted LSM3 or DHX16 72 hours before analysis.
